# Tandem Mass Tag (TMT)-based quantitative proteomics reveals potential targets associated with onset of Sub-clinical Mastitis in cows

**DOI:** 10.1038/s41598-020-66211-6

**Published:** 2020-06-09

**Authors:** Shveta Bathla, Anil Sindhu, Sudarshan Kumar, Shivam Kumar Dubey, Smaranika Pattnaik, Preeti Rawat, Alka Chopra, Ajay Dang, Jai Kumar Kaushik, Ashok Kumar Mohanty

**Affiliations:** 1 Animal Biotechnology Centre, ICAR-National Dairy Research Institute, Karnal, 132001 Haryana India; 2 Animal Physiology Division, ICAR-National Dairy Research Institute, Karnal, 132001 Haryana India; 3 Department of Biotechnology, Deenbandhu Chhotu Ram University of Science and Technology, Sonepat, 131039 Haryana India

**Keywords:** Protein-protein interaction networks, Diagnostic markers

## Abstract

Bovine milk is vital for infant nutrition and is a major component of the human diet. Bovine mastitis is a common inflammatory disease of mammary gland in cattle. It alters the immune profile of the animal and lowers the quality and yield of milk causing huge economic losses to dairy industry. The incidence of sub-clinical mastitis (SCM) is higher (25–65% worldwide) than clinical mastitis (CM) (>5%), and frequently progresses to clinical stage due to lack of sensitive and specific detection method. We used quantitative proteomics to identify changes in milk during sub-clinical mastitis, which may be potential biomarkers for developing rapid, non-invasive, sensitive detection methods. We performed comparative proteome analysis of the bovine milk, collected from the Indian hybrid cow Karan Fries. The differential proteome in the milk of Indian crossbred cows during sub-acute and clinical intramammary gland infection has not been investigated to date. Using high-resolution mass spectrometry-based quantitative proteomics of the bovine whey proteins, we identified a total of 1459 and 1358 proteins in biological replicates, out of which 220 and 157 proteins were differentially expressed between normal and infected samples. A total of 82 proteins were up-regulated and 27 proteins were down-regulated, having fold changes of ≥2 and ≤0.8 respectively. Among these proteins, overexpression of CHI3L1, LBP, GSN, GCLC, C4 and PIGR proteins was positively correlated with the events that elicit host defence system, triggering production of cytokines and inflammatory molecules. The appearance of these potential biomarkers in milk may be used to segregate affected cattle from the normal herd and may support mitigation measures for prevention of SCM and CM.

## Introduction

Mastitis is an inflammation of the mammary gland which is broadly classified into sub-clinical (no clinical symptoms of infection) and clinical mastitis (physiological symptoms present) on the basis of severity of infection. The sub-clinical infections are usually manifested by increases in somatic cell counts (SCC) and decrease in milk production. However, clinical infections are marked by changes in milk such as appearance of clots, flakes or watery texture, further accompanied by fever, depression and anorexia^1^. The detection of sub-clinical mastitis is difficult due to the absence of any visible indications, and remains the main cause of economic loss for the dairy industry because of its direct impact on highly reduced milk yield in the affected cows and non-acceptability of milk by consumers^2^. One of the major causes of mastitis in cattle is bacterial infection. Gram-positive contagious pathogens such as *Staphylococcus* may cause persistent, sub-clinical and clinical infection while Gram-negative coliform bacteria from the environment, such as *E. coli* and *Klebsiella*, frequently cause an acute inflammation response with severe clinical signs^3^. The bacterial load causes mammary gland damage, which reduces the number and activity of epithelial cells through disruption of alveolar cell integrity, sloughing of cells, and induced apoptosis. The invading bacteria produces toxins or byproduct which have a destructive impact on mammary gland cells. On the other hand, active host immune cells migrate into the mammary gland which leads to breakdown of the blood-milk barrier and causes epithelial cell death. Progressive cases of inflammation eventually render the udder unfit for milk production and inflict severe pain and suffering to the animals^4^. A large number of methods are in place to detect mammary gland inflammation which includes both direct and indirect approaches. However, the precise diagnosis of the sub-clinical form of mastitis is not possible until now as the early signs of inflammation are inconspicuous.

In the last few years, a lot of work has been done to unravel the host-pathogen interactions and related defensive molecular mechanisms of mastitis, aiming to specifically recognize and quantitate biomarkers at an early stage that indicates sub-clinical mastitis^5–7^. To date, no confirmatory method is available that could diagnose sub-clinical mastitis at an early stage with high accuracy and precision. Advances in mass spectrometry-based quantitative proteomics techniques, such as two-dimensional gel electrophoresis (2D-GE)^8^, label-free^9^ and labelled^10^ approaches, have been used to identify and quantify several host-specific milk proteins during mastitis which may be potential biomarker candidates for diagnosis of sub-clinical mastitis. Despite previous efforts by various groups, the available information on monitoring protein expression changes in milk during sub-clinical mastitis is inconclusive. A large number of factors govern the profile of the milk proteome. Therefore, comprehensive analysis of changes in the milk proteome during sub-clinical mastitis may increase our understanding of milk composition, mammary biology, and immune function in the mammary gland as well as identifying new biomarker targets for early detection of sub-clinical mastitis. Thus, application of the novel protein biomarkers in detection of sub-clinical mastitis can serve as a valuable tool in the development of new diagnostic tools for implementing control measures.

We hypothesized that the invading pathogen activates immunological and metabolic pathways which may alter the expression of proteins secreted in milk. To identify protein expression changes during sub-clinical and clinical infection, we used a Tandem Mass Tag TMT-based quantitative proteomic approach to identify potential biomarkers for early detection of sub-clinical mastitis.

## Results

We used high-throughput TMT mass spectrometry based relative quantitation of the whey proteome to identify differentially expressed proteins during healthy, sub-clinical and clinical infection. The somatic cell count (SCC) threshold in this study was: Healthy (7 × 10^4^–1 × 10^5^ cells/ml), SCM (2–5 × 10^5^ cells/ml) and CM (13–15 × 10^5^ cells/ml). Furthermore, California Mastitis Test (CMT) was done to support the SCC based classification (Supplementary Table 1). The differences in SCC of healthy and sub-clinical cases were highly distinct between the groups to avoid any misinterpretation. In dairy ruminants, SCC and CMT are typically used as an udder health index and as an inflammatory indicator to diagnose mastitis, because it represents the number of neutrophils in milk.

To some extent, the quality of milk is determined by its SCC. The significant increase of SCC in milk indicates poor quality of milk due to low level of lactose, protein, fat and relatively high pH, sodium, and chloride content (Supplementary Table 1). The information derived from SCC is often ambiguous because it is affected by factors other than mastitis, such as the season and the animal’s age, lactation period, diet, and other physiological conditions.

**Figure 1:**
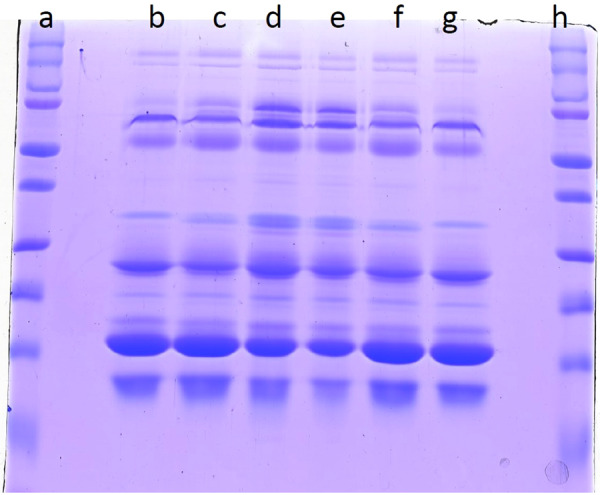
SDS Profile of whey prepared from healthy, sub-clinical and clinical mastitis affected animals: Lane a & h- protein marker (230–15 kDa), Lane b & e- Healthy pooled protein (n = 10 for each replicate), Lane c & f- SCM pooled protein (n = 10 for each replicate). Lane d & g- CM pooled protein (n = 10 for each replicate).

Careful analysis of the milk proteome during early onset of infection in cows may provide biomarkers that allow detection of sub-clinical mastitis. However, the existence of highly abundant proteins with a large dynamic range, such as caseins, pose a major challenge in the identification of less abundant proteins. Thus, caseins were removed by ultracentrifugation to enable maximum proteome coverage. The 1D gel profile of whey collected from healthy, sub-clinical and clinical mastitis milk is shown in Fig. 1.

### Protein identification and Relative quantification

**Figure 2:**
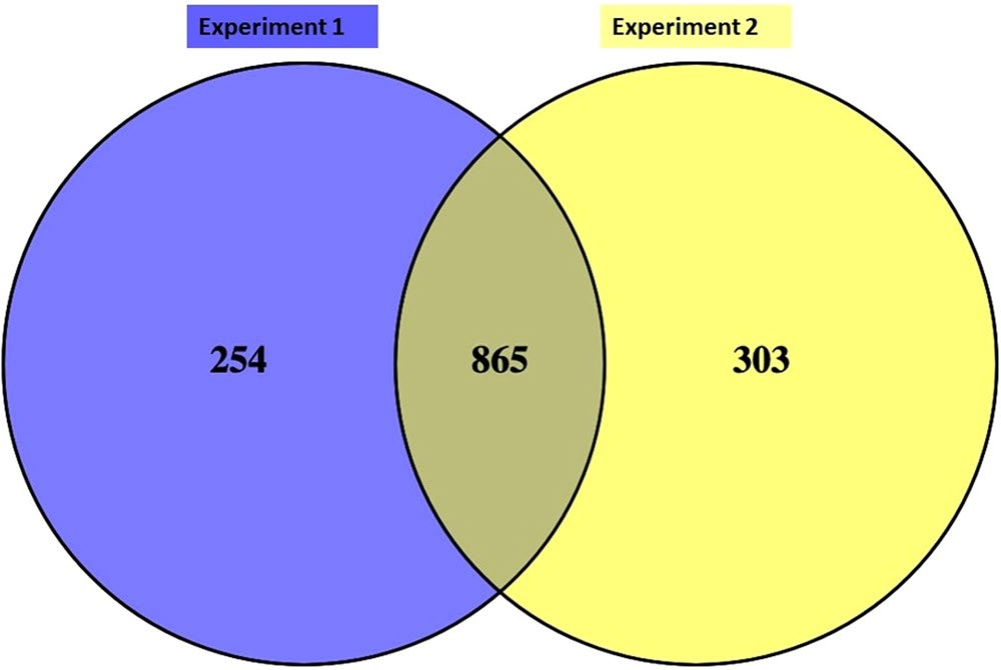
Venn diagram showing the comparison of whey proteins identified in biological replicates.

**Figure 3:**
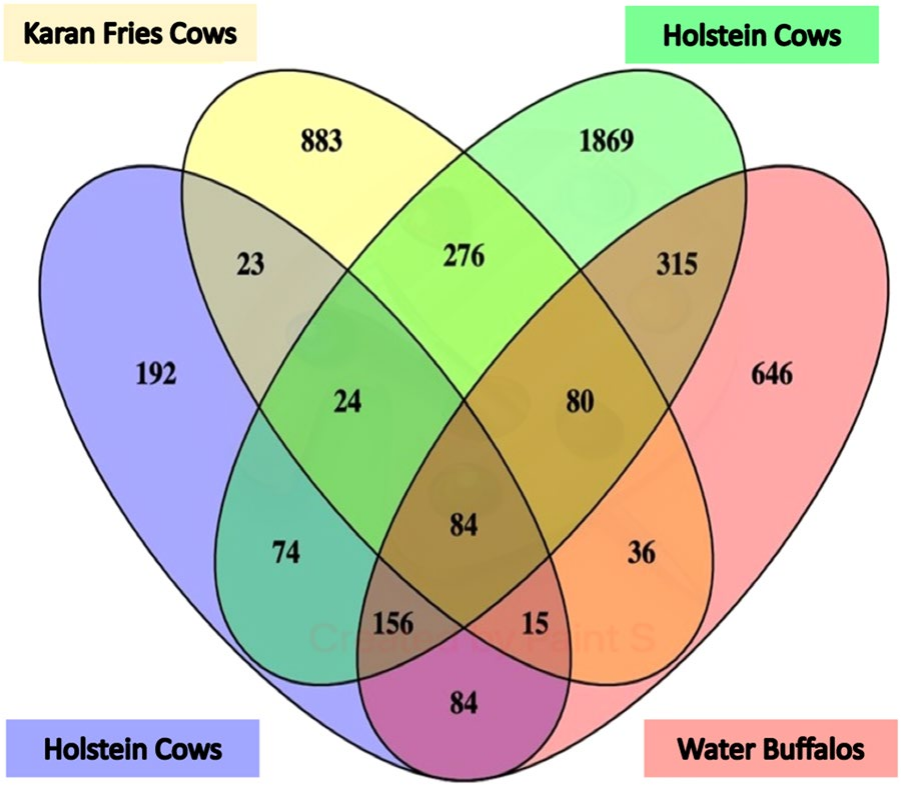
Venn diagram showing comparison of whey proteome of Karan Fries cows with previously reported data of Holstein cows and water buffalo^11–13^.

A 6-plex TMT-nLC-MS/MS analysis generated 1459 and 1358 proteins having ≥2 peptides with a false discovery rate (FDR) of 1% in biological replicates respectively (Supplementary File). The identified proteins were non-redundant with 95% confidence having a Mascot score >28. A total of 865 proteins were found in common between the biological replicates after removal of uncharacterized proteins; results of this study was also compared with the milk proteome of different breed of cow and buffalo such as Holstein and water buffalo as shown in Figs. 2 and 3.

Most of the identified proteins in both groups had molecular weights in the range of 1–800 kDa and pI in the range of 4.0–12.9 (Supplementary Figure 1). A total of 220 & 157 proteins were differentially expressed in both replicates out of which 90 common differentially expressed proteins (DEPs) having a fold change ≥ 2 & ≤0.8 (≥2 peptides) were selected for further bioinformatics analysis (Supplementary Table 2). The panel of proteins including Glutamate-cysteine ligase, lipopolysaccharide binding protein, Chitinase3, Gelsolin, and G protein-coupled receptors that showed enhanced expression in sub-clinical and clinical milk were validated by western blot (Supplementary Figure 2).

### Functional classification of differentially expressed proteins

**Figure 4:**
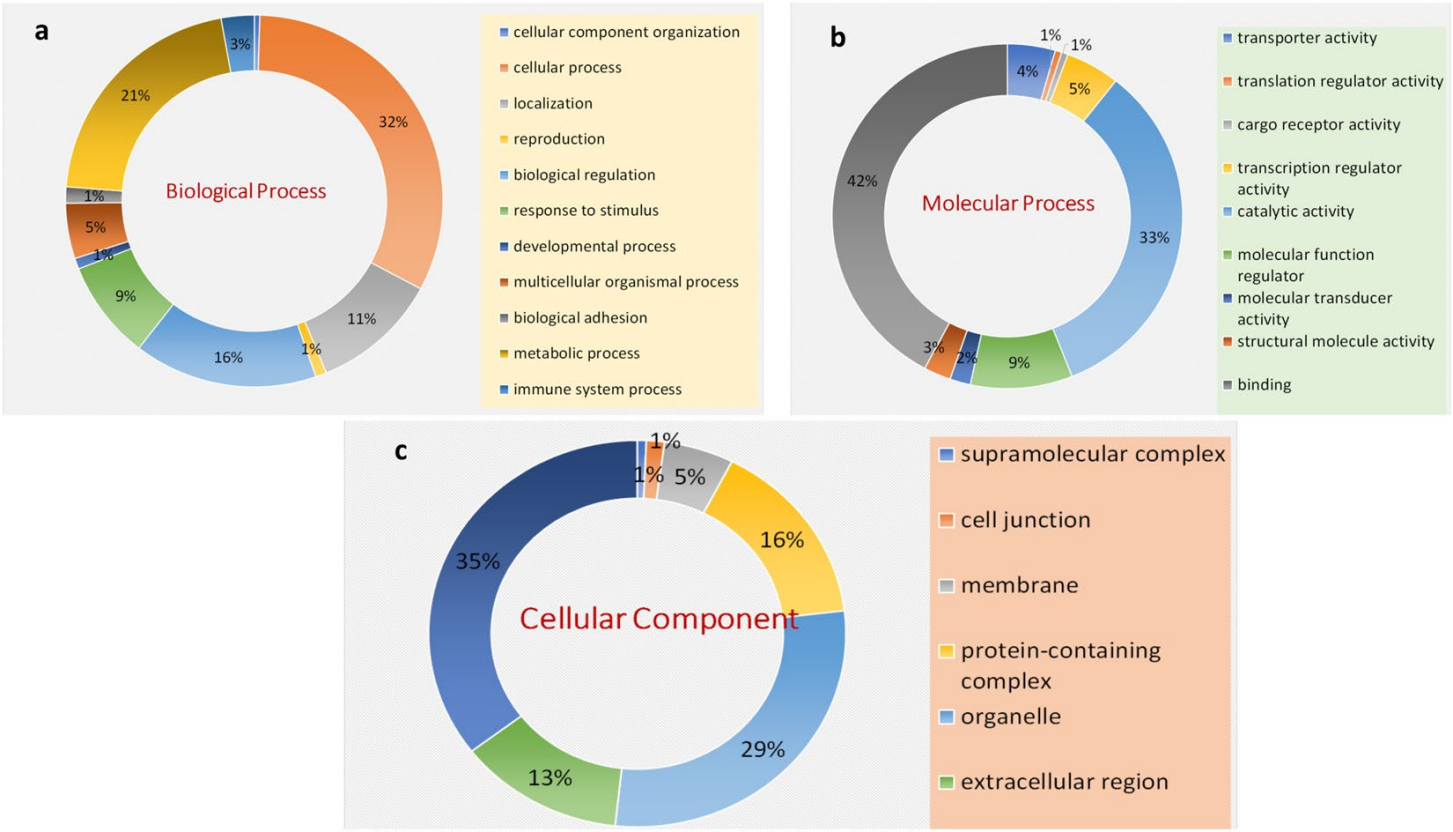
Gene Ontology classification of proteins on the basis of their involvement in(**a)** Biological process **(b)** Molecular function **(c)** Cellular component.

Total of common 90 DEP’s with an arbitrary fold change cut-off ≥2 & ≤0.8 in both replicates were classified into 11 categories according to their putative biological functions revealed by PANTHER Software Fig. 4(a). DEPs were involved in cellular process (32.00%), metabolic process (21.00%), response to stimulus (9.10%), biological regulation (16.70%), localization (9.30%), multicellular organism process (5.10%) and immunological response (3.0%). The other categories include biological adhesion (1.50%), developmental process (1.0%), reproduction (1%), and biogenesis (0.50%). On the basis of metabolic functions, DEPs were divided into 9 categories. Most proteins were involved in binding activity (42.10%) followed by catalytic activity (33.30%), molecular function regulator (9.40%), transcription regulator activity (5.0%), transport (4.40%). The remaining activities were signal transducing activity (2.60%), structural molecular activity (2.50%), molecular transducer activity (1.90%), followed by cargo receptor activity (0.60%) (Fig. 4c). Further, the PANTHER enrichment analysis of cellular location, biological, and metabolic processes was performed using the Fisher extract test with Bonferroni correction (P < 0.05) (Supplementary Table 3).

## Protein Interaction Network

**Figure 5:**
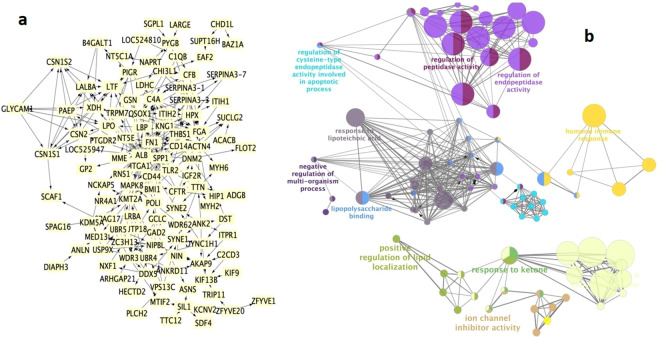
The protein-protein interaction and functional annotation: **(a)** Network generated with STRING 10.0 using combined score of (0.9–0.4) and visualized with Cytoscape 2.8.1 for DEPs. The PPI network with a high combined score constructed by 182 nodes and 877 edges with an average node degree of 13.5 with clustering coefficient 0.4–0.9. **(b)** Functional annotation of interacting hub was done using ClueGo.

Protein-protein interaction networks play important roles in biological process. A protein interaction map was generated using the publicly available program, the Search Tool for the Retrieval of Interacting Genes/Proteins (STRING) for understanding the altered protein-protein interaction networks in mastitis. Involvement of detected proteins in immune-pathophysiological pathways was visualized in Cytoscape software (Fig. 5a). The protein-protein interaction network of DEPs with the highest confidence scores (>9.0) was constructed with 182 nodes, 877 edges, and an average node degree of 13.5 with clustering coefficient 0.4. Each interaction has a combined score (between 0.4 and 0.9), which represents the reliability of the interaction between proteins.

**Figure 6:**
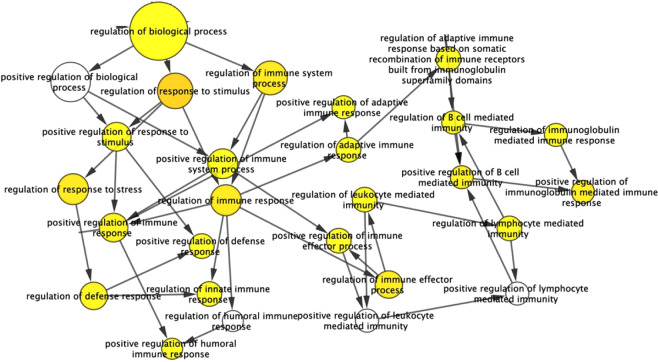
Enrichment analysis obtained by BINGO 2.44 software. Color bar in the right lower quadrant indicates level of significance from low (yellow) to high (orange). Statistical analysis was performed with a hypergeometrical test. A p-value of0.05 was considered significant.

By removing unconnected proteins and self-loops, proteins with higher connectivity in the network are referred to as hubs. The predicted modes of actions between hubs and their associated DEPs were revealed by ClueGo software (Fig. 5b) which was further enriched using Hypergeometric test and Benjamini & Hochberg false discovery rate correction in Bingo Software (Fig. 6).

**Figure 7:**
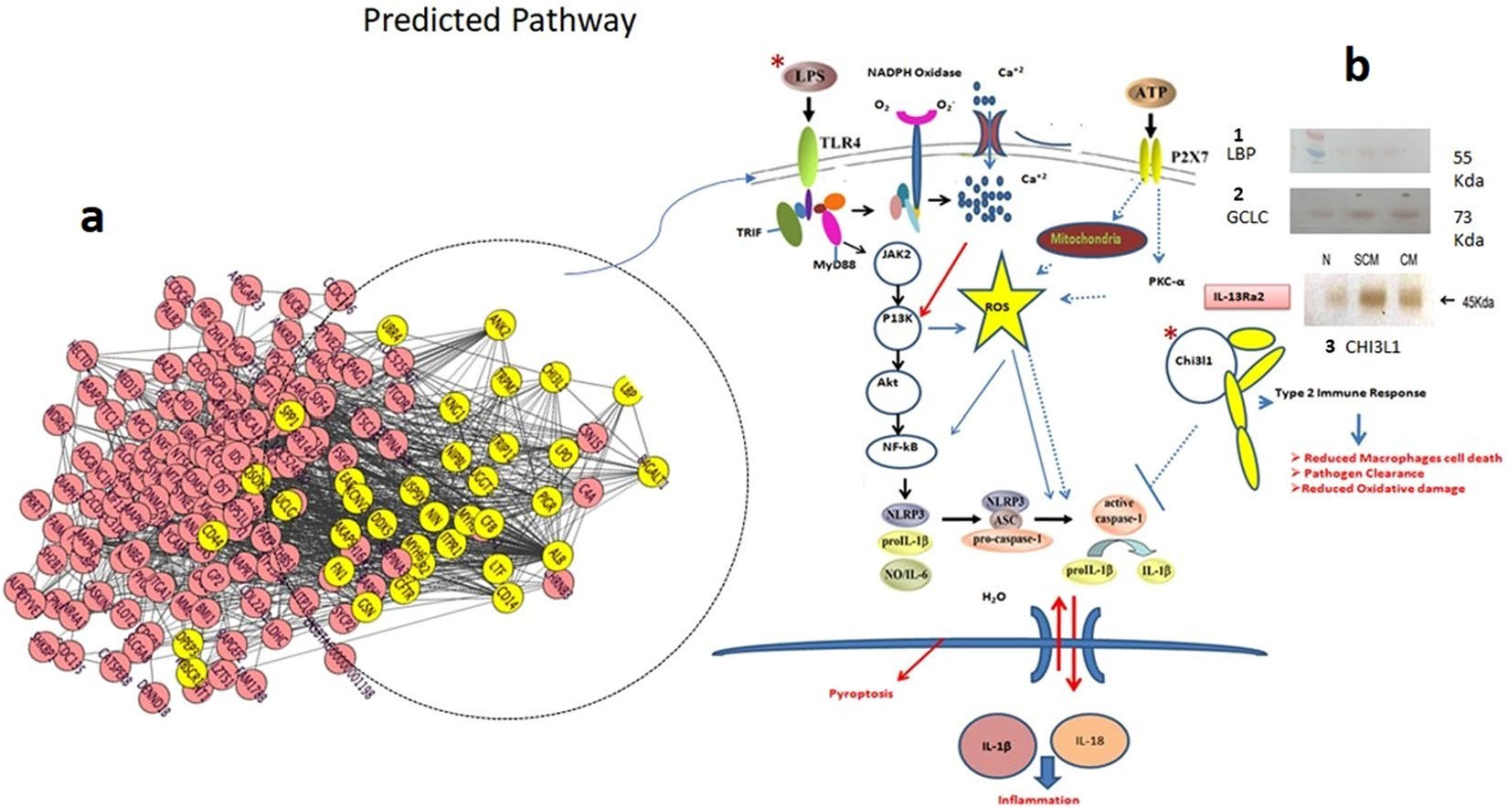
(**a**) The interacting proteins visualized using Cluster-viz software M-CODE algorithm (degree threshold-2, node score threshold-0.2, K-core threshold-2 and max depth-100). **(b)** Targets of predicated pathway were validated using Western blot: 1. LBP 2. GCLC 3.CHI3L1.

Using protein-protein interaction and pathway analysis, we have observed the strong enrichment of the inflammatory response (p = 8.1540E-8), wound healing response (p = 2.0747E-7), stress response (p = 9.7749E-7), positive regulation of immune response (p = 1.7342E-5), positive regulation of response to stimulus (p = 3.6834E-5), activation of plasma proteins involved in the acute inflammatory response (p = 6.5504E-5), complement activation (p = 6.5504E-5), defense response to bacterium (p = 5.1749E-4), and lactose biosynthetic process (p = 1.1476E-4). Furthermore, an interacting protein hub of target proteins was generated by Cluster viz software using M-CODE algorithm (Fig. 7a). The predicated role of identified targets in the activation of host resistance such as inflammation, pyroptosis, oxidative injury, and host tolerance was demonstrated and further validated by western blot (Fig. 7b).

## Discussion

Identification of protein repertoires in the milk of cows affected by mastitis is a valuable strategy for discovery of potential biomarkers for the possible development of novel diagnostic tests with increased accuracy for sub-clinical mastitis. In our study, we identified 1459 and 1358 proteins with a minimum of 2 peptides in biological replicates. A previously, few studies reported the 768, 1530, and 2971 whey proteins and whole milk of Holstein cows and water buffalo respectively^11–13^. However, 538 whey proteins overlapped with the previously catalogued whey proteins and suggesting possible influence of geography, lactation, feed, breed, or weather-related differences on protein expression^14–16^. Therefore, our results significantly expand the number of identified proteins in the bovine whey proteome and significantly contributes the novel targets for sub-clinical diagnosis. In the present investigation, DEPs with high significance such as CHI3LI, LPB, GCLC, GSN, and CD14 are actively associated with host-pathogen interaction and further activation of the immunological response.

### Pathogen-binding and activation of inflammasome

Increased expression of LPB protein during sub-clinical and clinical infection was observed. LPB a 452aa protein with a molecular mass of 60 kDa, is an acute-phase reactant produced during Gram-negative bacterial infections. It is synthesized mainly in the liver, pulmonary, and gastrointestinal epithelial cells. It acts as a carrier for LPS and helps to control LPS-dependent monocyte responses^17^.

The immune system has evolved to allow a host to control and eliminate the pathogen which is known as host resistance. The immune cells trigger finely tuned cellular responses for the host response by sensing the pathogen-associated molecular pattern (PAMPs) of bacterial origin. The recognition of pathogen-derived products such as lipopolysaccharide (LPS) by Toll-like receptor 4 (TLR4) and co-operative binding with CD14 and MD2 promotes the endocytosis of the LPS-bound TLR4 receptor complex. This TLR4 signaling complex further facilitates the interaction of TLR4 and TRIF, which triggers the nuclear translocation of NF-κB promoting the activation of IRF3 resulting in expression of type 1 interferon^18^. The LPS-induced endocytosis of TLR4 and subsequent TRIF-mediated IRF3 activation are highly dependent on CD14, which is a GPI-linked cell surface protein. It has been proposed that CD14 promotes the activation of Syk and phosphorylation of Cγ^2^, which is necessary for Ca^+2^ dependent endocytosis of TLR4^19^.

In our study, we observed altered expression of LPB (FC-**2**.0) and CD14 (FC-**1.7**) which reflects activation of the host immune response against infection. The expression of these proteins increased as infection worsened. The M-CODE algorithm based Cluster-viz software revealed the interacting partner of LBP, which is associated with activation of immunological responses (Fig. 7a).

The significant changes in expression of LBP, TLR, CH3L1 and CD14 during the early stages of infection make them suitable candidates for early infection diagnostic biomarkers for sub-clinical mastitis. Furthermore, these proteins were validated by western blot showing increased expression in the sub-clinical and clinical samples as compared to healthy samples. The pathogen-associated immune response also causes collateral damage to host tissues independent of the bacterial burden, which contributes to clinical symptoms during infection. The concept of preventing immune damage and promoting repair to host tissues during infection is known as host tolerance, disease tolerance, or tissue resilience. Therefore, some proteins act as silencers of the immune response, which facilitates a limited immune response that causes less host damage.

Relatedly, CHI3L1 plays a critical role in anti-pathogen response by augmenting bacterial killing while stimulating disease tolerance by controlling cell death, inflammation, and remodeling. It is expressed by a variety of cells including macrophages, neutrophils, and epithelial cells in the lung and digestive tract^20^. It has been demonstrated that CHI3L1 binds to interleukin-13 and IL-13Rα2 to form a heterodimer which leads to anti-apoptosis by subsequent activation of mitogen-activated protein kinase (MAPK) and AKT cellular signaling pathways^21^. This protein is also known to inhibit innate immune activation while promoting T helper type 2 (Th2) responses that can contribute to tissue healing and fibrosis. In our study, we found significantly increased expression of CHI3L1 during sub-clinical (FC-**4.05**) and clinical infection (FC-**9.99**) which depicts the activation of host resistance and host tolerance for infection. In the present study, increased expression of CHI3L1 suggests its role in the enhancement of host tolerance by reducing cell death, inflammation, and pyroptosis. This protein can be considered a potential diagnostic biomarker for sub-clinical infection which was further validated using western blot.

### Inflammation

Our study found the altered expression of Gelsolin (GSN) during sub-clinical infection. Previous studies suggest that GSN acts as an actin-scavenging protein which is responsible for depolymerization and capping of actin filaments, which are normally released into circulation upon cell death. In our data, increased expression of GSN can be considered as an indicator of inflammation that binds to LPS produced by bacteria and diminishes the activation of LPS and TLR-mediated inflammation process. The increased amount of plasma GSN suggests its role in wound healing and tissue modeling during infection^22^.

### Anti-oxidant activity

The study also revealed increased expression of glutamate-cysteine ligase (GCLC) during SCM (FC-**6.27)** and CM (FC-**8.43**), which is a key enzyme for the synthesis of glutathione. GSH is the main non-protein thiol in mammalian cells that participates in many critical cellular functions, including antioxidant defense and cell growth. It helps to deactivate the free radicals generated through immunological responses and maintain homeostasis^23^. In the present study, enhanced levels of GSH and enzymes related to its synthesis such GCLC and GS act as an indication of stress and infection in animals. It can be considered as a putative biomarker for sub-clinical infection in cows.

### Proteins involved in complement and antibody activation

The increased expression of C4 was observed in infected animals (SCM and CM) as compared to healthy animals. The complement component C4 plays a role in the activation of the classical and lectin pathways, leading to cleavage of C2, C3, and C5.

The C4 activates the complement pathway which results in the rapid clearance of bacteria by immune cells, and direct bacterial killing via large pore-forming complexes. C4 deficiency is associated with increased pneumococcal infection, and autoreactive IgA production and IgA kidney deposition in mice and humans. Thus, this finding suggests the involvement of C4 cascades proteins in the clearance of pathogen within infected cattle^24^.

Furthermore, understanding the molecular interplay between complement and bacteria is of great importance for future therapies of infectious and inflammatory diseases. In the current study, we observed enhanced expression of polymeric immunoglobulin receptor (PIGR) during sub-clinical infection and clinical infection. PIgs are made by plasma cells in the lamina propria underlying the epithelial barrier and transported across the epithelial barrier with the help of the polymeric immunoglobulin receptor (pIgR)^25^. The PspC–pIgR mediated pneumococcal uptake by host epithelial involving a concerted role of host cell cytoskeleton and signaling pathways has been previously demonstrated. The activation events contribute to cell membrane dynamics and promotes pneumococcal ingestion by host cells. However, the host endocytosis machinery involved in the pIgR-mediated pneumococcal uptake is not known^26^.

### Protein with miscellaneous functions

G protein-coupled receptors (GPCRs) comprise a superfamily of proteins capable of transducing a wide range of extracellular signals across the plasma membrane of the cell into discrete intracellular messages capable of regulating numerous, diverse cell functions^27^. The association of GPCR with various inflammatory diseases was demonstrated as these receptors mediates the flow of short chain fatty acids (SCFAs) such as acetate, propionate, and butyrate, which regulates neutrophil chemotaxis, T cell differentiation, activation, and subsequent cytokine production. We observed increased expression of G protein-coupled receptor 44 protein in sub-clinical and clinical mastitis affected animals as compared to healthy animals in our study. Therefore, GPR4 could be considered as a potential diagnostic biomarker and drug target for inflammatory diseases^28^.

Interestingly, we found increased expression of myosin in sub-clinical and clinical mastitis affected animals. Myosin is a molecular motor that provides force for cell movement via catalyzing hydrolysis of ATP and participates in a wide range of biological processes in many eukaryotic cells, such as cell adhesion, cell migration, cell division, and pinocytosis^29^.

The interaction of the heavy chain of cell surface myosin in human umbilical vein endothelial cells with surface glycoprotein of thrombocytopenia syndrome virus (SFTSV) was identified by LC-MS/MS. Considering the involvement of myosin in many disease pathologies, it can be considered as therapeutic target for diagnosis and prevention of pathological conditions^30^.

The increased expression of APP and antimicrobial proteins during mammary gland infection has been reported^10^. However, no significant changes were observed in expression of APP and antimicrobial proteins in the current study. However, unaltered expression of protein indicates that the immune system was not active enough to produce significant levels of anti-microbial peptides, leading to inefficient eradication of the bacteria at the site of infection.

## Conclusion

Clearly, several challenges still remain regarding the identification and accurate quantification of biomarkers of the host response in bovine milk for sub-clinical mastitis due to the inherent biological variability of animals within same breed or different breeds, along with environmental effects such as season, diet, and physiological condition of animal. Nonetheless, the high-throughput proteomics approach with two technical replicates during mass spectrometric identification and quantitation generates clear differences between healthy and sub-clinical mastitis milk samples, which can serve as candidate molecules in the development of a diagnostic kit. Further, the wide spectrum of proteins perturbed during mastitis involved in immunity, metabolism and adhesion will help derive clues for its control and prevention. The data presented here provides an important panel of candidate proteins which may be used as diagnostic biomarkers for sub-clinical mastitis.

## Material and Methods

### Milk collection

**Figure 8:**
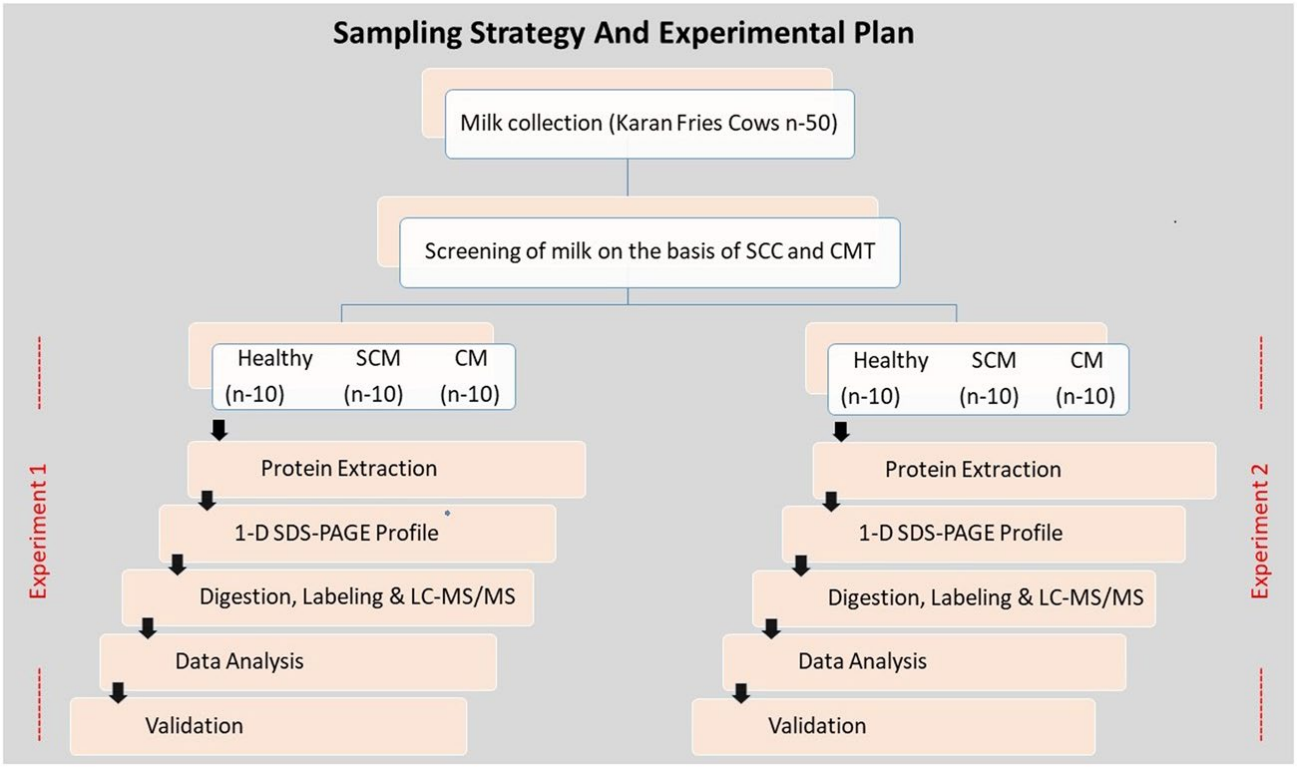
Sampling strategy and experimental plan.

The milk samples were collected from different udder quarters of 50 Karan Fries cows comprising of 200 quarters. A pool of samples (n = 10) was prepared in duplicate out of 20 biological replicates for each group (healthy, sub-clinical and clinical mastitis) for subsequent SDS PAGE and proteomic analyses (Fig. 8). Briefly, teats were cleaned and disinfected using 70% ethanol (vol/vol). The initial three streams of milk were discarded and approximately 50–100 mL of milk was collected into a sterile plastic tube without preservative.

### SCC determination

2–3 ml of milk was used for somatic cell count (SCC) within two hours of milk collection. Using SCC, the milk samples were categorized into healthy (7 × 10^4^–1 × 10^5^ cells/ml), sub-clinical (2–5 × 10^5^ cells/ml) and clinical (13–15 × 10^5^ cells/ml). Further, CMT was used for reconfirmation of SCC.

### Milk processing and whey preparation

50 mL of collected milk was defatted by centrifuging at 5000 x g for 20 min at 4 °C and subsequently were stored at −80 °C for further analysis. For whey preparation, defatted milk samples were thawed at 4 °C and ultra-centrifuged at 60,000 x g for 2 h at 4 °C so that samples had a pellet of casein micelles at bottom, a fat layer on the top, and dilipidated whey supernatant in the middle. Whey samples were collected in fresh 50 mL centrifuge tubes^14^.

### SDS profile of whey proteins

The protein concentration of whey samples were determined with a Bradford assay (Biorad, USA) and 20 μg of protein from each sample was resolved on 4% stacking and 12% separating acrylamide-bis acrylamide gel using 100 V over 3 h using miniVE Vertical Electrophoresis System (GE Healthcare, USA). The gels were then stained with Coomassie Brilliant blue G (Sigma, Canada) overnight, de-stained for 2 h and scanned with an EPSON scanner (GE, Healthcare, USA).

### Protein digestion and TMT labeling

100 µg of protein sample was taken from pooled (n = 10) healthy, sub-clinical, and clinical mastitis samples and dissolved in dissolution buffer (0.5 M triethylammonium-bicarbonate, pH 8.5), denatured with 2% SDS followed by reduction of protein using 50 mM tris-(2 carboxyethyl) phosphine (TCEP) at 60 °C for 1 h. Alkylation of cysteine residues was performed using 20 mm IAA in the dark for 30 min followed by tryptic digestion (Promega, 1:20) at 37 °C overnight. The peptide derived from healthy, sub-clinical and clinical samples were labelled with 126, 127, or 128 TMT reagent respectively using TMT 6 Plex (Thermo) according to the manufacturer’s protocol. Peptides were labelled with respective tags and incubated for 2 h, quenched and vacuum centrifuged to dryness.

### Fractionation of labelled peptides using b-RPLC

The pooled labelled peptides were loaded onto C18 column (4.6 × 250 mm, C18, 5 µm, Grace, USA) on the Dionex, quaternary U-HPLC system (Ultimate3000, Thermo, USA) with UV detection at 214 nm. HPLC solvents A and B consisted of 10 mM TEABC (pH 8.5) and 10 mM TEABC in 90% ACN, respectively. The peptides were resolved at 25 °C with a flow rate of 1 mL/min and continuous gradient elution (5–100% ACN) from the column over a period of 81 min. The gradient was set up with 2–60% solvent B (65 minutes) followed by 60–100% solvent B (10 minute) and 100% solvent B (5 minute). Ninety-six time-based fractions were collected, which were pooled to twentyfour individual fractions by mixing the most hydrophobic with the most hydrophilic and vacuum dried samples for both groups. The dried samples were acidified in 20 µl of 0.1% FA and desalted using C18 Ziptips (Millipore Billerica MA, USA). The desalted fractions were stored at −80 °C following vacuum centrifugation^31^.

### ESI- LC-MS/MS analysis

The fractions were analyzed on maxis-HD (Bruker, Bremen, Germany) interfaced with Nano-LC (Bruker, Bremen, Germany). Peptides were initially enriched on a reversed phase liquid chromatography (RPLC) pre-column (2 cm, 5 µ – 100 Ǻ), followed by separation on an analytical column (15 cm, 3 µ – 100 Ǻ) (Agilent). The peptides were sprayed using nano electro spray emitter tip of 10 µm (Bruker, Bremen, Germany). The solvent system used includes 0.1% aqueous formic acid as solvent A and 100% acetonitrile, 0.1% formic acid as solvent B. The peptides were loaded on the trap column using 97% solvent A, followed by separation on the analytical column using a linear gradient of 5–30% solvent B for 70 min at a constant flow rate of 0.400 µL/min. The spray voltage and heated capillary temperature were set to 2.0 kV and 220 °C, respectively. The data was acquired in data-dependent acquisition mode subjecting the six most intense ions in each survey scan to MS/MS analysis within the *m/z* range of 400–2200. The precursor fragmentation was carried out using collision-induced dissociation (CID) as the activation method. The precursor ions selected for MS/MS fragmentation were excluded after every three spectra. The absolute threshold for precursor ions per 1000 summations was 1200 counts^32^.

### Data analysis

The MS/MS data were searched against the UniProt *Bos taurus* database for peptide identification and quantification using Mascot 2.1 (Matrix Science, London, U.K.) search engine in Protein Scape Software 3.2 (Bruker). The search parameters for identification and relative quantification of proteins were as follows: peptides were considered to be tryptic, one mis-cleavage allowed, carboxyamidomethylation at cysteine residue, TMT labeling at peptide N-terminus and lysine residue were considered as fixed modification, whereas oxidation at methionine was considered as variable. The mass tolerance for the precursor ions was 0.1 Da and that for fragment ions was 0.1 Da. To eliminate false positives, 1% FDR was applied at both protein and peptide level. The relative abundance of proteins was reported as the median value, calculated as the ratio of peak intensity for peptides labelled with TMT channels for a given protein. The TMT ratio was derived for each peptide with reporting intensity above the threshold (S/N > 1). The MS data has been deposited to the Proteome X change consortium with the PRIDE partner repository with the database identifier PXD014922^33^.

### Bioinformatics analysis and Network construction

Protein classification on the basis of their molecular function, related biological process, and cellular component was performed using Protein Analysis Through Evolutionary Relationships (PANTHER). To ensure proper analysis of the complex interactions between the proteins, the networks were constructed using STRING v10.0 with high confidence (0.70)^34^. To further understand the biological significance of the enrichment terms and associated metabolic pathways, we used enrichment/depletion with two-sided hypergeometric distribution tests, with Bonferroni adjustment, followed by a *p-value* significance level cut-off of ≤0.05 for the terms and the groups creation in Cytoscape 2.8.1 software^35^ with plug-in ClueGO^36^.

### Western blot analysis

The protein was separated by a 12% SDS-PAGE gel and then transferred onto a PVDF membrane. After blocking overnight at room temperature in TBST (20 mM Tris-HCl, 140 mM NaCl, pH 7.5, 0.05% Tween-20) containing 3% BSA, the membrane was incubated separately with three primary antibody: anti-TRMP7, anti-LBP, and anti-CH3LI (Cloud-Clone Corp. & Santa Cruz Biotechnology, TX USA) at dilutions of 1:500, 1:2000, and 1:500 respectively overnight at 4 °C. Then membranes were washed 3 times with TBST and incubated with horseradish peroxidase-conjugated secondary antibody (diluted 1:1000, Sigma Aldrich, USA) for 1 h at room temperature. Visualization of the immunoreactive proteins was accomplished using DAB staining.

### Statistical analysis

The Fisher extract test with Bonferroni correction (P < 0.05) was performed for gene ontology enrichment in PANTHER software. For ClueGO and BiNGO, statistical analysis was performed using two-sided hypergeometric distribution tests with Benjamini and Hochberg false discovery rate (FDR)-correction at P < 0.05.

### Ethics approval and consent to participate

Ethics Statement Approval of Institute Animal Ethics (IAEC) committee was not required because the experiment did not involve any invasive procedures for animal experiments. The milk samples were collected from the dairy herd of National Dairy Research Institute (NDRI), Karnal which is a public funded research institute under the Indian Council of Agricultural Research, Government of India. The milk is routinely collected every day from the lactating animals for routine care and research purposes. The animals were maintained under expert veterinary supervision. NDRI has all the necessary permits for the housing and care of animals for scientific purposes vide registration no. 1705/GO/ac/13/CPCSEA 3rd July, 2013 duly approved by Ministry of Environment and Forest, Govt. of India (Web site: http://envfor.nic.in).

## Supplementary information

Supplementary Information1.

Supplementary Information2.

## Data Availability

Data are freely available for the readers.
