# Supplementary material for: Tandem Mass Tag (TMT)-based quantitative proteomics reveals potential targets associated with onset of Sub-clinical Mastitis in cows
Source: Sci Rep. 2020 Jun 9;10:9321. doi: 10.1038/s41598-020-66211-6 (PMC7283279; doi:10.1038/s41598-020-66211-6)
Supplement: Supplementary file 2 — Supplementary Information2. [file 41598_2020_66211_MOESM2_ESM.pdf]

Tandem Mass Tag (TMT)-based quantitative proteomics reveals potential targets associated with on-set of Sub-Clinical Mastitis in cows

Shveta Bathla[1,3], Anil Sindhu[3], Sudarshan Kumar[1]*, Shivam Kumar Dubey[1], Smaranika Pattnaik[1], Preeti Rawat[1], Alka Chopra[1], Ajay Dang[2], Jai Kumar Kaushik[1], Ashok Kumar Mohanty[1]*

[1]Animal Biotechnology Centre, ICAR-National Dairy Research Institute, Karnal, 132001, Haryana, India

[2]Animal Physiology Division, ICAR-National Dairy Research Institute, Karnal, 132001, Haryana, India

[3]Department of Biotechnology, Deenbandhu Chhotu Ram University of Science and Technology, Sonepat, 131039, Haryana, India

***Corresponding Authors:** ashokmohanty1@gmail.com, kumarsudershan@gmail.com

Supplementary Table 1: Sample Collection and Milk Composition Analysis

S.No	Animal No	Quarter	SCC	pH	EC	Fat	SNF	Density	Lactose	Solids	Protein	Temp	FP	CMT Score
1	7669	LFQ	$233x10^3$	6.62	4.76	3.20	9.00	28.74	5.02	0.66	2.94	28.6	-0.575	1
		RFQ	$142x10^3$	6.61	4.74	4.4	8.69	26.57	4.86	0.63	2.83	28.4	-0.561	1
		LHQ	$\geq 1500x10^3$	6.6	4.95	3.73	8.76	27.38	4.89	0.64	2.86	29.5	-0.561	3
		RHQ	$351x10^3$	6.61	4.73	4.34	8.64	26.42	4.83	0.63	2.81	29.9	-0.556	2
2	7383	LFQ	$1287\ x10^3$	6.57	5.32	4.20	8.14	24.60	4.55	0.59	2.63	29.1	-0.521	3
		RFQ	$\geq 1500x10^3$	7.09	12.73	0.55	7.31	24.29	4.09	0.53	2.33	30.2	-0.445	3
		LHQ	$\geq 1500x10^3$	6.74	6.07	4.88	7.8	22.71	4.37	0.56	2.5	29.6	-0.5	3
		RHQ	$\geq 1500x10^3$	6.89	8.54	2.29	7.87`	25.06	4.40	0.57	2.53	30.7	-0.491	3
3	7696	LFQ	$93x10^3$	6.51	5.63	3.37	8.42	26.34	4.70	0.61	2.73	30	-0.535	0
		RFQ	$1489x10^3$	7.22	10.19	2.59	8.00	25.32	4.47	0.58	2.58	32.1	-0.501	3
		LHQ	$132x10^3$	6.57	5.67	3.05	8.42	26.60	4.71	0.61	2.73	30.8	-0.533	1
		RHQ	$1489x10^3$	6.73	8.05	2.87	7.81	24.38	4.37	0.56	2.51	32.3	-0.49	3

4	7697	LFQ	$1489 x 10^3$	6.67	7.04	3.19	8.44	26.56	4.72	0.61	2.74	33.5	-0.535	3
		RFQ	$286 x 10^3$	6.60	5.36	3.50	8.38	26.08	4.68	0.61	2.72	33.5	-0.533	1
		LHQ	$322 x 10^3$	6.67	5.43	4.95	8.09	23.78	4.53	0.58	2.60	33.4	-0.521	2
		RHQ	$93 x 10^3$	6.67	5.28	3.76	8.32	25.64	4.65	0.60	2.69	33.2	-0.53	0
5	7488	LFQ	$405 x 10^3$	6.73	6.66	2.97	8.69	27.71	4.85	0.64	2.83	29.8	-0.551	2
		RFQ	$1489 x 10^3$	6.96	8.27	2.64	8.74	28.16	4.88	0.64	2.85	30.5	-0.553	3
		LHQ	$111 x 10^3$	6.76	6.12	2.81	8.66	27.72	4.84	0.63	2.82	29.6	-0.548	1
		RHQ	$1489 x 10^3$	6.90	8.82	3.17	8.60	27.21	4.81	0.63	2.80	30.5	-0.546	3
6	7525	LFQ	$\leq 70 x 10^3$	6.60	5.13	5.85	7.34	20.16	4.12	0.51	2.33	31.5	-0.474	0
		RFQ	$\leq 70 x 10^3$	6.59	5.00	6.60	7.08	18.55	3.98	0.49	2.33	31.4	-0.459	0
		LHQ	$279 x 10^3$	6.59	5.19	3.79	7.87	23.89	4.41	0.57	2.53	31.3	-0.50	2
		RHQ	$\leq 70 x 10^3$	6.59	5.51	3.80	7.89	23.92	4.41	0.57	2.53	31.0	-0.50	0
7	7685	LFQ	$\leq 70 x 10^3$	6.7	6.0	4.19	7.86	23.52	4.40	0.56	2.52	31.3	-0.501	0
		RFQ	$\leq 70 x 10^3$	6.73	6.01	3.50	8.03	24.70	4.45	0.57	2.58	31.9	-.503	0

		LHQ	$82x10^3$	6.7	5.68	2.16	8.62	28.10	4.82	0.63	2.81	31.1	-0.542	0
		RHQ	$\leq 70x10^3$	6.74	5.64	4.02	7.96	24.03	4.45	0.57	2.56	31.8	-0.507	0
8	7728	LFQ	$1489x10^3$	7.00	10.45	5.16	7.61	21.74	4.26	0.54	2.43	31.5	-0.489	3
		RFQ	$139x10^3$	6.71	6.21	1.61	8.69	28.82	4.85	0.64	2.84	31.7	-0.543	1
		LHQ	$261x10^3$	6.77	5.93	5.66	7.39	20.15	4.15	0.52	2.35	31.4	-0.476	1
		RHQ	$1489x10^3$	7.22	10.58	4.53	7.36	21.29	4.15	0.52	2.34	31.7	-0.468	3
9	7686	LFQ	$1489x10^3$	6.86	5.60	2.34	8.54	27.63	4.77	0.62	2.78	31.7	-0.537	3
		RFQ	$\leq 70x10^3$	6.77	5.34	2.89	8.47	26.92	4.73	0.62	2.75	31.7	-0.536	0
		LHQ	$163x10^3$	6.74	5.45	2.19	8.77	28.65	4.90	0.64	2.86	31.8	-0.552	1
		RHQ	$\leq 70x10^3$	6.73	5.11	3.82	8.33	25.63	4.66	0.60	2.70	31.9	-0.531	0
10	7234	LFQ	$1489x10^3$	6.91	8.12	8.01	6.04	13.37	3.40	0.40	1.84	26.2	-0.393	3
		RFQ	$1141\ x10^3$	7.14	8.88	6.99	6.63	16.47	3.73	0.45	2.06	29.7	-0.429	3
		LHQ	$1489x10^3$	7.12	8.57	6.60	6.35	15.70	3.57	0.43	1.96	28.9	-0.408	3
		RHQ	$1489x10^3$	7.28	10.19	8.54	5.33	10.18	3.01	0.34	1.58	29.2	-0.346	3

11	7453	LFQ	≤70x10^3	6.81	5.65	1.35	9.10	30.16	5.08	0.67	2.99	28.9	-0.570	0
		RFQ	≤70x10^3	6.64	5.32	2.14	8.87	29.09	4.95	0.65	2.90	29.4	-0.559	0
		LHQ	1489x10^3	6.81	6.27	1.83	8.88	29.35	4.94	0.65	2.90	28.9	-0.557	3
		RHQ	1489x10^3	6.60	6.15	2.68	8.68	27.92	4.85	0.64	2.83	28.4	-0.549	3
12	7436	LFQ	1489x10^3	6.56	5.37	3.13	8.48	26.75	4.74	0.62	2.75	28.0	-0.538	3
		RFQ	1489x10^3	6.56	7.43	3.99	7.74	23.20	4.33	0.55	2.48	27.1	-0.491	3
		LHQ	1489x10^3	4.95	6.57	3.34	8.55	26.86	4.78	0.62	2.78	27.3	-0.544	3
		RHQ	1489x10^3	7.23	10.86	2.98	8.29	26.15	4.63	0.60	2.68	24.3	-0.52	3
13	7305	LFQ	1475x10^3	6.93	8.64	10.27	4.84	6.90	2.75	0.29	1.39	27.3	-0.314	3
		RFQ	305x10^3	6.95	9.11	7.08	6.11	14.38	3.44	0.41	1.87	29.8	-0.394	2
		LHQ	431x10^3	6.94	8.55	9.34	5.35	9.63	3.03	0.34	1.58	28.4	-0.351	2
		RHQ	1489x10^3	7.08	10.94	4.44	7.12	20.44	4.03	0.50	2.25	28.1	-0.451	3
14	7624	LFQ	1489x10^3	6.95	7.75	5.34	7.54	21.33	4.22	0.53	2.40	30.9	-0.485	3
		RFQ	314x10^3	6.68	6.64	3.33	7.87	24.23	4.40	0.57	2.53	31.1	-0.497	2

		LHQ	$1489x10^3$	6.96	10.50	5.42	7.85	22.49	4.40	0.56	2.51	31.1	-0.507	3
		RHQ	$258x10^3$	6.72	6.18	3.60	8.03	24.64	4.49	0.58	2.59	31.1	-0.509	1
15	7226	LFQ	$485x10^3$	6.59	5.31	7.05	7.41	19.47	4.16	0.52	2.35	31.9	-0.485	2
		RFQ	$100x10^3$	6.66	4.81	3.68	8.98	28.28	5.02	0.66	2.94	32.5	-0.576	1
		LHQ	$311x10^3$	6.71	4.85	4.41	8.72	26.66	4.87	0.63	2.84	32.4	-0.562	2
		RHQ	$293x10^3$	6.72	5.13	3.68	9.05	28.55	5.05	0.66	2.96	31.9	-0.581	1
16	7261	LFQ	$97x10^3$	6.60	4.33	4.84	8.74	26.42	4.89	0.64	2.84	32.6	-0.567	0
		RFQ	$167x10^3$	6.59	4.64	4.30	8.80	27.07	4.92	0.64	2.87	32.5	-0.567	1
		LHQ	$750x10^3$	6.57	4.37	7.23	7.85	21.05	4.40	0.55	2.51	32.6	-0.518	2
		RHQ	$1489x10^3$	6.58	4.57	5.75	8.49	24.70	4.75	0.61	2.75	32.7	-0.555	3
17	7664	LFQ	$342x10^3$	6.58	4.97	3.82	8.43	26.06	4.71	0.61	2.73	32.3	-0.539	2
		RFQ	$293x10^3$	6.60	5.12	2.84	8.74	28	4.88	0.64	2.85	31.2	-0.554	1
		LHQ	$754x10^3$	6.62	5.52	2.68	8.79	28.31	4.91	0.64	2.87	32.6	-0.556	3
		RHQ	$1489x10^3$	6.64	5.79	2.34	8.92	29.10	4.98	0.66	2.92	32.8	-0.563	3

18	7377	LFQ	$1489x10^3$	6.46	6.65	3.31	8.56	29.62	4.72	0.62	2.78	32.5	-0.544	3
		RFQ	$883x10^3$	6.51	5.58	4.11	8.56	29.78	4.85	0.63	2.83	33.1	-0.558	3
		LHQ	$1153x10^3$	6.55	6.35	4.17	8.40	25.61	4.69	0.61	2.72	33	-0.538	3
		RHQ	$1489x10^3$	6.57	6.45	3.96	8.38	25.71	4.69	0.61	2.71	32.9	-0.536	3
19	7343	LFQ	$160x10^3$	6.54	5.25	4.92	8.18	24.15	4.57	0.59	2.63	33.1	-0.527	1
		RFQ	$191x10^3$	6.55	5.23	5.43	7.99	23.01	4.47	0.57	2.56	33.2	-0.517	1
		LHQ	$520x10^3$	6.55	5.16	5.30	8.22	24	4.60	0.59	2.65	33.4	-0.532	2
		RHQ	$1489x10^3$	6.55	5.49	5.22	8.30	24.40	4.64	0.60	2.68	33.5	-0.538	3
20	7338	LFQ	$142x10^3$	6.50	5.39	2.40	8.60	27.81	4.80	0.63	2.80	33.5	-0.542	1
		RFQ	$82x10^3$	6.52	6.18	1.72	8.46	27.83	4.73	0.62	2.75	33.3	-0.528	0
		LHQ	$683x10^3$	6.49	5.73	2.70	8.40	26.81	4.70	0.61	2.73	33.5	-0.530	2
		RHQ	$1489x10^3$	6.59	5.49	3.14	8.74	27.76	4.88	0.64	2.85	30.2	-0.556	3

21	7303	LFQ n6	$86x10^3$	6.47	4.97	3.06	9.09	29.21	5.08	0.67	2.98	30.3	-0.580	0
		RFQ	$1227x10^3$	6.57	5.61	3.14	8.74	27.76	4.88	0.64	2.85	30.2	-0.556	3
		LHQ	$516x10^3$	6.54	5.06	3.26	8.90	28.31	4.97	0.65	2.91	31.0	-0.568	2
		RHQ	$289x10^3$	6.52	4.75	3.29	9.14	29.19	5.10	0.67	2.99	30.4	-0.585	1
22	7543	LFQ	$132x10^3$	6.57	5.89	1.30	8.86	29.73	4.95	0.65	2.90	31.2	-0.553	1
		RFQ	$<70x10^3$	6.6	5.95	0.73	9.04	30.87	5.04	0.67	2.97	31.1	-0.562	0
		LHQ	$265x10^3$	6.58	5.61	1.43	8.98	30.08	5.01	0.66	2.94	31.6	-0.562	1
		RHQ	$<70x10^3$	6.60	5.60	1.59	8.91	29.68	4.97	0.66	2.92	31.6	-0.558	0
23	7640	LFQ	$x10^3$	Not working										
		RFQ	$<70x10^3$	6.56	5.01	2.53	8.86	28.72	4.95	0.65	2.89	30.7	-0.560	0
		LHQ	$<70x10^3$	6.54	5.01	3.31	8.52	26.77	4.76	0.62	2.77	31.3	-0.54	0
		RHQ	$<70x10^3$	6.52	5.04	2.90	8.65	27.59	4.83	0.63	2.82	31.1	-0.548	0
24	7634	LFQ	$1489x10^3$	6.70	9.11	2.60	7.71	24.18	4.31	0.55	2.47	29.6	-0.482	3
		RFQ	$1489x10^3$	6.91	8.94	4.70	6.87	19.26	3.86	0.48	2.16	29.9	-0.435	3

		LHQ	$1489x10^3$	7.01	9.02	3.71	7.17	21.20	4.02	0.51	2.27	30.3	-0.451	3
		RHQ	$633x10^3$	6.87	6.26	4.26	7.74	23	4.34	0.55	2.48	30.5	-0.493	2
25	7100	LFQ	$<70x10^3$	6.67	5.61	0.87	8.57	28.93	4.78	0.63	2.79	30.2	-0.531	0
		RFQ	$1287x10^3$	6.63	5.84	2.08	8.20	26.52	4.58	0.60	2.65	30.2	-0.512	3
		LHQ	$<70x10^3$	6.60	5.60	0.91	8.56	28.86	4.78	0.63	2.79	30	0.530	0
		RHQ	$x10^3$	Not working										
26	7121	LFQ	$114x10^3$	6.49	4.09	1.6	10.19	31.86	5.26	0.7	3.61	-0.6	30.4	1
		RFQ	$118x10^3$	6.44	3.98	1.78	10.15	31.58	5.24	0.7	3.6	-0.598	30.5	1
		LHQ	$1325x10^3$	6.43	4.52	1.83	10.09	31.33	5.21	0.69	3.58	-0.595	30.4	3
		RHQ	$1216x10^3$	6.45	4.49	1.8	10.1	31.36	5.21	0.69	3.58	-0.595	30.6	3
27	7144	LFQ	$591x10^3$	6.45	5.34	3.72	9.2	26.36	4.73	0.61	3.24	-0.544	29.4	2
		RFQ	$174x10^3$	6.45	4.47	4.72	9.24	25.68	4.74	0.61	3.25	-0.553	28.7	1

		LHQ	$908x10^3$	6.46	7.48	3.46	8.85	25.18	4.53	0.59	3.11	-0.518	29	2
		RHQ	$408x10^3$	6.49	7.18	3.97	8.72	24.26	4.46	0.57	3.06	-0.511	29.6	2
28	7184	LFQ	$362x10^3$	6.43	5	4.72	8.45	22.61	4.31	0.55	2.96	-0.497	30.8	2
		RFQ	$1500x10^3$	6.45	7.18	3.85	8.44	23.28	4.31	0.55	2.96	-0.491	30.7	3
		LHQ	$139x10^3$	6.51	4.33	4.4	8.75	24.04	4.48	0.57	3.07	-0.516	30.8	1
		RHQ	$357x10^3$	6.46	4.84	5.02	8.37	22.07	4.27	0.54	2.93	-0.493	31	2
29	7236	LFQ	$100x10^3$	6.41	5.59	2.35	9.33	27.95	4.79	0.63	3.29	-0.545	30.7	1
		RFQ	$675x10^3$	6.4	5.31	2.2	9.31	27.99	4.78	0.63	3.29	-0.542	30.5	2
		LHQ	$377x10^3$	6.38	5.28	2.24	9.46	28.54	4.86	0.64	3.34	-0.553	30.6	2
		RHQ	$114x10^3$	6.34	5.27	2.19	9.44	28.51	4.85	0.64	3.34	-0.552	30.7	1
30	7069	LFQ	$1247x10^3$	6.38	7.08	2.77	8.81	25.6	4.51	0.58	3.1	-0.511	28.3	3
		RFQ	$1489x10^3$	6.34	4.77	3.96	9.01	25.39	4.62	0.6	3.17	-0.532	27.3	3

		LHQ	$1489x10^3$	6.4	6.11	3.75	8.6	23.97	4.39	0.56	3.02	-0.502	26.8	3
		RHQ	$1468x10^3$	6.44	5.28	3.03	9	26.13	4.61	0.6	3.17	-0.526	26.5	3
31	7771	LFQ	$286x10^3$	6.4	4.87	5.76	8.62	22.45	4.41	0.56	3.02	-0.515	30.8	2
		RFQ	$368x10^3$	6.38	4.35	10.86	6.67	10.8	3.35	0.38	2.29	-0.402	30.9	2
		LHQ	$325x10^3$	6.38	4.87	4.73	9.02	24.85	4.63	0.6	3.17	-0.538	30.9	2
		RHQ	$334x10^3$	6.41	4.81	5.47	8.73	23.11	4.47	0.57	3.06	-0.521	31	2
32	7258	LFQ	$1091x10^3$	6.10	6.52	3.43	8.57	24.11	4.37	0.56	3.01	-0.498	27.3	1
		RFQ	$305x10^3$	6.20	5.27	2.37	9.28	27.74	4.77	0.62	3.28	-0.541	26.8	2
		LHQ	$1489x10^3$	6.19	6.77	2.41	9.26	27.61	4.75	0.62	3.27	-0.540	26.8	3
		RHQ	$1489x10^3$	6.26	5.91	2.68	9.36	27.81	4.81	0.63	3.30	-0.549	27.2	3
33	7233	LFQ	$1489x10^3$	8.66	7.55	2.83	8.23	23.29	4.19	0.54	2.89	-0.472	25.8	3
		RFQ	$362x10^3$	8.39	4.24	3.46	9.07	26.04	4.65	0.6	3.19	-0.533	24.9	2

		LHQ	$422x10^3$	8.12	4.13	3.63	9.01	25.69	4.62	0.6	3.17	-0.53	24.7	2
		RHQ	$311x10^3$	9.31	4.27	3.97	8.94	25.11	4.58	0.59	3.14	-0.527	24.9	2
34	7075	LFQ	$485x10^3$	7.91	7.97	4.73	7.64	19.46	3.87	0.48	2.66	-0.44	25.2	2
		RFQ	$74x10^3$	7.8	5.01	1.43	9.95	31.08	5.13	0.68	3.52	-0.582	26.8	0
		LHQ	$47x10^3$	7.57	5.82	2.11	9.58	29.1	4.93	0.65	3.39	-0.561	26.1	0
		RHQ	$<70x10^3$	7.43	4.77	2.17	9.91	30.35	5.11	0.68	3.51	-0.584	28.5	0
35	7266	LFQ	$1489x10^3$	7.35	6.88	3.04	9.7	28.82	5	0.66	3.43	-0.575	26.8	3
		RFQ	$322x10^3$	7.29	4.76	5.49	9.26	25.16	4.76	0.61	3.26	-0.559	27.7	2
		LHQ	$1489x10^3$	7.22	6.78	4.49	9.3	26.09	4.78	0.62	3.27	-0.556	28.2	3
		RHQ	$240x10^3$	7.18	4.88	4.47	9.66	27.53	4.98	0.65	3.41	-0.582	28.8	2
36	7069	LFQ	$1489x10^3$	7.15	6.11	3.84	9.42	27.08	4.84	0.63	3.32	-0.56	28.1	3
		RFQ	$325x10^3$	7.09	4.28	4.58	9.7	27.58	5	0.65	3.42	-0.585	28	2

		LHQ	$293x10^3$	6.99	4.38	4.17	9.86	28.56	5.09	0.67	3.48	-0.594	28.5	2
		RHQ	$195x10^3$	6.95	4.21	5.03	9.58	26.76	4.93	0.64	3.38	-0.579	29	2
37	7068	LFQ	$244x10^3$	6.09	5.01	3.24	10.1	30.22	4.83	0.69	3.57	0.605	31	2
		RFQ	$240x10^3$	6.47	5	3.14	10.03	30.04	4.72	0.69	3.58	0.599	31.3	2
		LHQ	$174x10^3$	6.48	4.75	3.24	10.11	30.25	5.19	0.69	3.55	-0.605	31.2	1
		RHQ	$188x10^3$	6.49	4.73	3.16	10.14	30.44	5.06	0.69	3.59	-0.607	31.2	1
38	7180	LFQ	$342x10^3$	6.55	5.33	5.22	8.82	23.65	4.93	0.58	3.1	-0.526	31.6	3
		RFQ	$558x10^3$	6.62	4.83	3.9	9.52	27.45	4.28	0.64	3.36	-0.568	31.4	3
		LHQ	$118x10^3$	6.65	4.1	4.95	9.45	26.33	4.87	0.63	3.33	-0.571	31.3	1
		RHQ	$160x10^3$	6.63	4.29	5	9.27	25.63	4.77	0.62	3.26	-0.557	31.3	1
39	7403	LFQ	$254x10^3$	6.59	5.35	3.82	9.32	26.72	4.79	0.62	3.28	-0.553	31.5	2
		RFQ	$181x10^3$	6.6	4.94	4.38	9.27	26.07	4.76	0.62	3.26	-0.553	31.4	1

		LHQ	177x10^3	6.58	4.62	4.67	9.27	25.83	4.76	0.62	3.26	-0.555	31.5	1
		RHQ	132x10^3	6.58	4.46	4.3	9.48	26.96	4.88	0.62	3.34	-0.567	31.7	1
40	7346	LFQ	320x10^3	6.07	5.5	5.16	8.65	23.04	4.42	0.56	3.03	-0.514	28.8	2
		RFQ	216x10^3	6.08	4.77	4.77	8.73	22.71	4.47	0.57	3.06	-0.524	29	2
		LHQ	216x10^3	6.11	5.13	5.48	8.75	23.17	4.48	0.57	3.07	-0.522	27.6	2
		RHQ	153x10^3	6.12	5.22	5.03	8.84	23.88	4.53	0.58	3.1	-0.526	29	1
41	7023 (3 teat present)	LFQ	1489x10^3	5.7	10.89	4.51	7.46	18.95	3.77	0.47	2.6	-0.427	28.3	3
		RFQ	-	-	-	-	-	-	-	-	-	-	-	
		LHQ	1489x10^3	5.72	9.23	6.75	7.28	16.44	3.67	0.44	2.52	-0.425	28.1	3
		RHQ	1489x10^3	5.78	12.02	2.18	7.77	22.02	3.94	0.5	2.72	-0.437	26.7	3
42	7032	LFQ	600x10^3	6	6.18	2.43	9.4	28.16	4.83	0.63	3.32	-0.55	27.5	2
		RFQ	<70x10^3	6.01	4.18	3.1	9.99	29.89	5.15	0.68	3.53	-0.596	28	0

		LHQ	637x10^3	6.01	7.1	2.51	9.1	26.93	4.67	0.61	3.21	-0.53	27.7	2
		RHQ	247x10^3	6.02	4.79	3.21	9.69	28.66	4.99	0.66	3.42	-0.576	27.1	2
43	7057	LFQ	78x10^3	5.39	4.87	3.74	9.45	27.31	4.86	0.64	3.33	-0.562	28.1	0
		RFQ	<70x10^3	5.52	4.59	4.57	9.33	26.15	4.79	0.62	3.28	-0.558	28.9	0
		LHQ	<70x10^3	5.61	4.61	3.94	9.5	27.32	4.89	0.64	3.35	-0.566	27.3	0
		RHQ	1489x10^3	5.63	12.04	2.03	7.83	22.37	3.97	0.5	2.74	-0.44	28.3	3
44	7108 (2 teat present)	LFQ	-	-	-	-	-	-	-	-	-	-	-	
		RFQ	983x10^3	6.01	5.08	4.13	9.7	27.95	5	0.65	3.42	-0.582	29.9	2
		LHQ	-	-	-	-	-	-	-	-	-	-	-	
		RHQ	700x10^3	6	7.73	3.41	9.04	25.98	4.64	0.6	3.18	-0.531	30	2
45	7192	LFQ	1489x10^3	6.08	6.39	6.11	8.21	20.56	4.18	0.52	2.87	-0.487	28	3
		RFQ	82x10^3	6.09	4.55	4.76	9.21	25.55	4.73	0.61	3.24	-0.551	28.8	0
		LHQ	<70x10^3	6.07	4.37	4.27	9.49	27.02	4.88	0.64	3.35	-0.568	28.6	0
		RHQ	<70x10^3	6.06	4.17	5.13	9.22	25.28	4.74	0.61	3.24	-0.554	28.6	0

46	7207	LFQ	<70x10^3	5.84	8.3	4.15	8.62	23.75	4.41	0.56	3.03	-0.506	28.7	0
		RFQ	261x10^3	5.86	5.52	3.07	9.74	29.98	5.02	0.66	3.44	-0.578	27.5	2
		LHQ	1489x10^3	5.81	6.24	1.91	9.98	30.8	5.15	0.68	3.53	-0.587	28.1	3
		RHQ	1489x10^3	5.82	6.9	1.95	9.84	30.25	5.07	0.67	3.48	-0.578	28.4	3
47	7270	LFQ	<70x10^3	5.79	4.15	4.45	9.15	25.57	4.7	0.61	3.22	-0.545	29.9	0
		RFQ	261x10^3	5.83	4.19	4.54	9.22	25.75	4.73	0.61	3.24	-0.55	30.2	2
		LHQ	1489x10^3	5.87	5.96	4.72	8.51	22.86	4.35	0.55	2.98	-0.501	30	3
		RHQ	1489x10^3	5.94	9.1	2.86	8.09	22.71	4.11	0.52	2.84	-0.462	29.4	3
48	7263	LFQ	139x10^3	6.01	4.05	4.51	9.46	26.73	4.87	0.63	3.34	-0.568	26.2	1
		RFQ	142x10^3	6.01	4	4.86	9.38	26.13	4.82	0.63	3.3	-0.564	28.3	1
		LHQ	198x10^3	6.03	4.36	4.33	9.44	26.77	4.85	0.63	3.33	-0.565	28.2	1
		RHQ	1489 x10^3	6.05	4.22	4.93	9.24	25.54	4.75	0.61	3.25	-0.555	27.4	3

49	7269 (3 teat present)	RFQ	$100x10^3$	5.82	4.69	1.47	10.78	34.29	5.59	0.75	3.83	-0.641	29.7	1
		LHQ	$107x10^3$	5.79	4.7	2.84	10.27	31.2	5.31	0.71	3.64	-0.614	29.7	1
		RHQ	$74x10^3$	5.75	4.68	1.64	10.68	33.76	5.53	0.74	3.79	-0.635	29.8	0
50	7319	LFQ	$<70x10^3$	5.99	4.58	3.49	9.63	28.18	4.96	0.65	3.4	-0.573	26.6	0
		RFQ	$<70x10^3$	5.94	4.79	3.72	9.4	27.12	4.83	0.63	3.32	-0.558	26.6	0
		LHQ	$<70x10^3$	5.95	4.95	3.93	9.31	26.59	4.78	0.62	3.28	-0.553	26.7	0
		RHQ	$<70x10^3$	5.98	6.12	3.23	8.96	25.81	4.59	0.6	3.16	-0.524	26.3	0

Supplementary Figure 1: Distribution of proteins on the basis of MW and pI

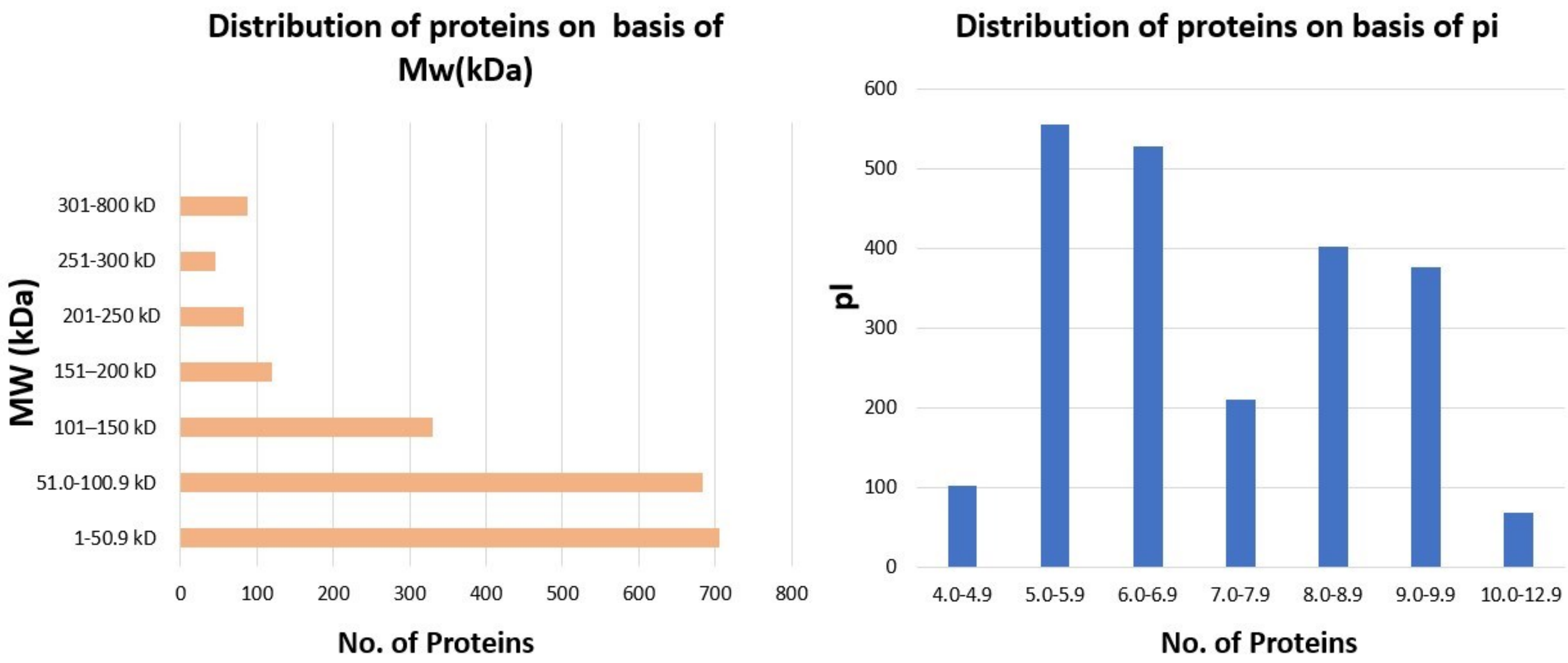

Supplementary Table 2: List of Differentially Expressed Proteins

Sr. No	Protein	Gene Symbol	Peptides	Experiment 1		Experiment 2	
				FC (SCM vs N)	FC (CM vs N)	FC (SCM vs N)	FC (CM vs N)
1	Hypothertical Protein		4	22.7	19.65		
2	Chitinase-3-Like Protein 1	CHI3L1	7	4.05	9.99	5.27	10.01
3	Glutamate--Cysteine Ligase Catalytic Subunit	GCLC	2	6.27	8.43	7.72	7.49
4	Glycogen Phosphorylase, Brain Form	PYGB	2	6.10	7.9	8.10	7
5	Transmembrane Channel-Like Protein 2	TMC2	2	6.06	7.47	7.6	6.5
6	Low-Density Lipoprotein Receptor-Related Protein 2-Like, Partial	LRP2	3	5.13	17.3	6.3	16.79
7	Nephrocystin-3	NPHP3	2	4.31	1.58	5.35	1.78
8	Protein-Coupled Receptor 44	GPR44G	2	3.77	17.70	3.79	16.9
9	Uncharacterized Protein	EPPK1	3	3.62	3.36	3.61	3.47

10	Leucine Zipper Putative Tumor Suppressor 2	LZTS2	3	3.61	5.97	3.89	5.47
11	Cytosolic 5'-Nucleotidase	NT5C1A	2	3.5	1.48	3.67	1.69
12	Dysferlin-Like	FER1L6	2	3.30	4.78	3.9	4.54
13	Uncharacterized Protein	SART1	2	3.25	3.34	4.25	4.34
14	Aldehyde Dehydrogenase Family 16 Member A1	ALDH16A 1	2	3.22	1.27	3.67	1.57
15	Histone-Lysine N-Methyltransferase Setmar	SETMAR	2	3.18	6.1	3.89	5.87
16	Myosin-7	MYH7	4	3.15	4.24	3.2	4.67
17	Cathelicidin-6	CATHL6	4	3.15	11.23	3.5	10.87
18	Leucine Rich Repeat And Coiled-Coil Domain Containing 1	LRRCC1	2	3.05	1.01	3.52	1.2
19	Bifunctional Udp-N-Acetylglucosamine 2Epimerase/N-Acetylmannosamine Kinase	GNE	2	2.94	3.08	3.84	3.67
20	186 Kda Protein	LTBP1	2	2.68	4.78	2.89	4.35

21	Uncharacterized Protein	ARHGAP1 1A	2	2.65	2.36	2.45	2.89
22	Mediator Of Rna Polymerase Ii Transcription Subunit 12	MED12	2	2.52	1.3	2.9	1.67
23	Cation Channel, SpermAssociated, Beta	CATSPER B	3	2.43	2.32	2.66	2.89
24	Rab Gdp Dissociation Inhibitor Beta	GDI2	6	2.42	3.11	2.9	3.47
25	Uncharacterized Protein	MAP4K1	2	2.41	2.63	2.78	2.52
26	Gelsolin	GSN	15	2.37	4.21		
27	Cation-Transporting P5-AtpaseLike	ATP13A4	2	2.27	1.85	2.49	2.1
28	Renin Receptor	ATP6AP2	2	2.24	3.5	2.47	3.67
29	Protein Diaphanous Homolog 3	DIAPH3	3	2.23	2.16	2.10	2.46
30	Dynein, Axonemal, Heavy Chain 5-Like	LOC10029 8754	10	2.03	3.37		
31	Lipopolysaccharide-Binding Protein	LBP	3	2.02	2.82	3.1	8.5

32	Uncharacterized Protein	C4A	16	2	4.82	1.4	2.2
33	Uncharacterized Protein	AKAP10	2	2.22	2.62	2.36	2.9
34	Temporarily Assigned Gene Name Family Member (Tag163)-Like	TNS3	2	2.19	1.19	2.4	2.1
35	Neuropilin- And Tolloid-Like Protein 2	NETO2	2	2.15	3.54	2.1	4.1
36	Zinc Finger And Btb Domain Containing 16	ZBTB16	2	2.14	3.03	2.4	3.4
37	Uncharacterized Protein	ZNF276	2	2.10	1.84	2.4	1.64
38	Soform 1 Of Active Breakpoint Cluster Region-Related Protein	ABR I	2	2.10	0.91	2.5	1.1
39	Stau2 Protein	STAU2	2	2.08	4.44	2.5	1.43
40	Uncharacterized Protein	CADPS2	3	2.03	1.63	2.4	2.74
41	Leucine-Rich Repeat Transmembrane Protein Flrt3	FLRT3	4	1.97	2.38	2.1	2.6
42	Ring Finger Protein 40	RNF40	3	1.97	2.37	2.05	2.67
43	Adp-Ribosylation Factor-Like 13B	ARL13B	2	1.97	2.3	2.2	2.1

44	Uncharacterized Protein	NFKB2	2	1.94	3.36	2.46	3.54
45	Synaptonemal Complex Protein2	SYCP2	3	1.92	2.15	2.12	2.4
46	Isoform Long Of Polymeric Immunoglobulin Receptor	PIGR	46	1.91	3.68	1.6	2.53
47	Uncharacterized Protein	CCNL1	2	1.90	2.31	2.1	2.51
48	Uncharacterized Protein	#######	2	1.89	3.38	2.1	3.4
49	Peptidyl-Prolyl Cis-Trans Isomerase C	PPIC	3	1.88	1.11	2.1	1.21
50	Uncharacterized Protein	LTBP2	2	1.88	0.99	2.1	1.1
51	Uncharacterized Protein	GCC1	2	1.88	2.09	2.15	2.2
52	39S Ribosomal Protein L37, Mitochondrial	MRPL37	3	1.81	2.54	2.1	2.5
53	Atpase, Class V, Type 10A	ATP10A	2	1.81	1.29	2.15	1.5
54	Uncharacterized Protein (Fragment)	STXBP5	2	1.81	2.62	2.2	2.4
55	Heat Shock 70 Kda Protein 13	HSPA13	3	1.80	1.56	2.1	2.7
56	Cingulin	CGN	3	1.79	2.08	2.1	2.2
57	Uncharacterized Protein	AFF3	3	1.79	3.67	2.15	3.4

58	Profilin-1	PFN1	3	1.78	2.49	1.9	2.6
59	Igll1 Protein	IGLL1	34	1.76	1.81	1.92	2.05
60	Advillin	AVIL	2	1.76	7.6	1.91	6.9
61	Troponin T Fast Skeletal Muscle Type	TNNT3	2	1.76	3.06	1.83	3.2
62	Tbc1 Domain Containing Kinase Isoform 3	TBCK	4	1.75	1.89	1.79	1.91
63	Uncharacterized Protein	BMP2	2	1.75	3.38	1.89	3.5
64	Isoform Short Of Beta-1,4Galactosyltransferase 1	B4GALT1	8	1.74	1.75		
65	Igl@ Protein	IGL@	52	1.74	1.40	2.05	2.1
66	Mitogen-Activated Protein Kinase 6	MAPK6	4	1.64	1.83		
67	Osteopontin	SPP1	22	1.60	4.08	1.05	1.72
68	Fibrinogen Alpha Chain	FGA	16	1.53	1.30	1.6	1.7
69	Thrombospondin-1	THBS1	19	1.46	2.48	1.4	3.33
70	Inter-Alpha-Trypsin Inhibitor Heavy Chain H1	ITIH1	4	1.41	1.75	2.7	2.3

71	Collagen,TypeXxii,Alpha 1-Like	COL22A1	2	1.39	1.50		
72	Fibronectin	FN1	7	1.05	1.32		
73	Isoform Lmw Of Kininogen-2	KNG1	15	0.98	0.91	1.2	2.05
74	Isoform Lmw Of Kininogen-1	KNG1	14	0.98	0.91	1.05	1.85
75	Beta-Lactoglobulin	PAEP	64	0.95	0.63	1.05	1.62
76	Uncharacterized Protein	DYNC1H1	6	0.79	0.4	0.72	0.51
77	Serpin A3-4	SERPINA3-3	18	0.78	1.00	0.71	1.1
78	Uncharacterized Protein	SERPINA3-8	10	0.78	1.00	0.76	1.05
79	Uncharacterized Protein	CHD2	2	0.78	1.77	0.75	1.87
80	Uncharacterized Protein	CD44	2	0.77	1.29	0.73	1.39
81	Dead (Asp-Glu-Ala-Asp) Box Polypeptide 5 Isoform 1	DDX5	2	0.76	1.12	0.72	1.22
82	Nucleolin	NCL	5	0.74	1.73	0.71	1.79
83	Uncharacterized Protein	LBR	2	0.70	0.74	0.71	0.79

84	Putative Malate Dehydrogenase 1B	MDH1B	3	0.63	1.62		
85	Uncharacterized Protein	GAD2	2	0.61	1.05	0.63	1.62
86	Uncharacterized Protein	MYO18A	5	0.60	2.31	0.63	2.6
87	Uncharacterized Protein	CLASP2	2	0.57	1.1	0.61	0.89
88	Tripeptidyl-Peptidase 1	TPP1	2	0.54	0.88	0.56	0.91
89	Uncharacterized Protein	NEK8	3	0.53	5.45	0.59	5.6
90	Filamin-A-Interacting Protein 1	FILIP1	3	0.31	1.2	0.35	1.4
91	Transcription Factor 5-Like	E2F5E2F	2	0.25	0.8	0.29	0.84
92	Centromere Protein N	CENPN	2	0.13	2.81	0.21	2.88

Supplementary Table 3: Enriched Processes revealed By Panther

S.No	Enriched biological process	log p value	Fold Enrichment
1	Maintenance of mitochondrion location	6.25077213	76.15
2	Maintenance of organelle location	5.11311704	34.61
3	Acute-phase response	4.44360665	21.76
4	Acute inflammatory response	3.92884404	15.23
5	Maintenance of location in cell	3.55090066	11.72
6	Maintenance of location	3.19219417	9.14
7	Humoral immune response	3.16349873	8.96
8	Inflammatory response	2.36737107	5.16
9	Microtubule-based process	1.96716861	3.91
10	Cell cycle process	1.78659636	3.45
11	Cytoskeleton organization	1.78240856	3.44
12	Cell cycle	1.56559718	2.96
13	Positive regulation of developmental process	1.41142625	2.66
14	Positive regulation of multicellular organismal process	1.33913738	2.53
15	Multi-organism process	1.28095631	2.43
16	Response to stress	1.22650853	2.34
17	Macromolecule localization	1.22032995	2.33
18	Organelle organization	1.04963077	2.07
19	Cellular component organization	0.97819563	1.97
20	Transport	0.94110631	1.92

21	Cellular component organization or biogenesis	0.94110631	1.92
22	Establishment of localization	0.93357264	1.91
23	Localization	0.93357264	1.91
24	Cellular process	0.50589093	1.42
25	Biological regulation	0.45417589	1.37
26	Biological process	0.25096157	1.19

S.No	GO molecular function	log p value	Fold Enrichment
1	Endopeptidase inhibitor activity	-5.9748294	7.07
2	Endopeptidase regulator activity	-5.6510878	6.85
3	Drug binding	-18.395516	2.54
4	Carbohydrate derivative binding	-24.630566	2.48
5	Anion binding	-28.381338	2.4
6	ATP binding	-12.505304	2.4
7	Adenyl ribonucleotide binding	-12.331656	2.39
8	Adenyl nucleotide binding	-12.302212	2.39
9	Small molecule binding	-16.60964	2.16
10	Purine ribonucleoside triphosphate binding	-9.7894615	2.15
11	Purine ribonucleotide binding	-10.087648	2.14
12	Ribonucleotide binding	-10.032212	2.13
13	Purine nucleotide binding	-10.014196	2.13
14	Nucleotide binding	-7.4985048	1.99
15	Nucleoside phosphate binding	-7.4985048	1.99
16	Ion binding	-11.574017	1.62
17	Protein binding	-7.166697	1.61
18	Binding	-13.996109	1.39
19	Transmembrane signaling receptor activity	-4.8868329	0.79

S.No	GO cellular component complete	log p value	Fold Enrichment
1	Ciliary plasm	-6.4050693	10.06
2	Axoneme	-6.4050693	10.06
3	Melanosome	-7.7894615	9.37
4	Pigment granule	-7.7894615	9.37
5	Midbody	-9.9231399	7.04
6	Plasma membrane bounded cell projection cytoplasm	-6.4803575	6.92
7	Extracellular space	-31.992772	4.88
8	Extracellular region part	-31.042958	4.12
9	Extracellular region	-39.114676	3.57
10	Cytoskeletal part	-9.7894615	2.56
11	Cytoplasmic vesicle	-5.72547	2.42
12	Intracellular vesicle	-5.7027499	2.41
13	Cytoskeleton	-10.396293	2.38
14	Vesicle	-5.4422223	2.33
15	Protein-containing complex	-14.000831	1.91
16	Endomembrane system	-5.0978878	1.83
17	Intracellular non-membrane-bounded organelle	-6.4173477	1.78
18	Non-membrane-bounded organelle	-6.4050693	1.78
19	Cytoplasmic part	-6.9301604	1.52
20	Organelle part	-5.4868125	1.48
21	Intracellular organelle part	-4.8911076	1.48
22	Cytoplasm	-6.9231399	1.44
23	Organelle	-10.124214	1.42

Supplementary Figure 2: Validation of protein targets using Western Blot

CHI3l1

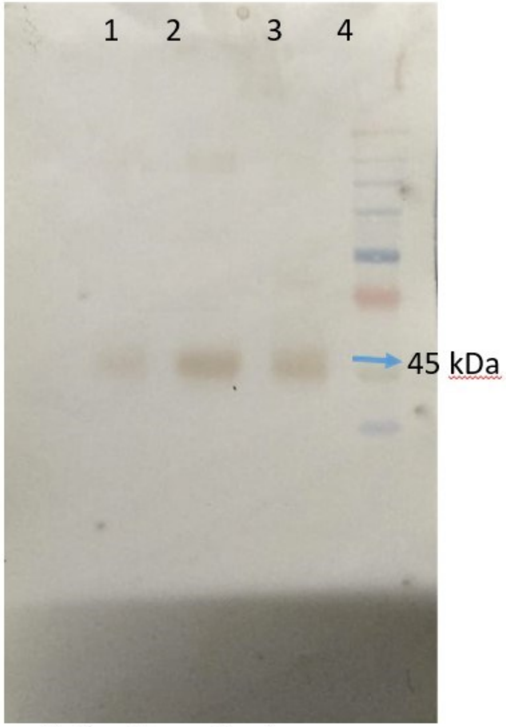

1-Healthy animal
2- SCM
3-CM
4- Marker

LBP

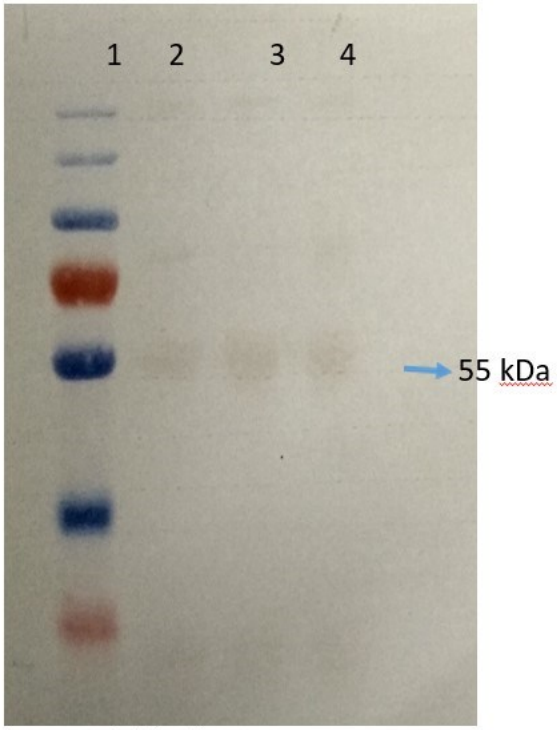

1-Marker
2-Healthy animal
3- SCM
4-CM

GCLC

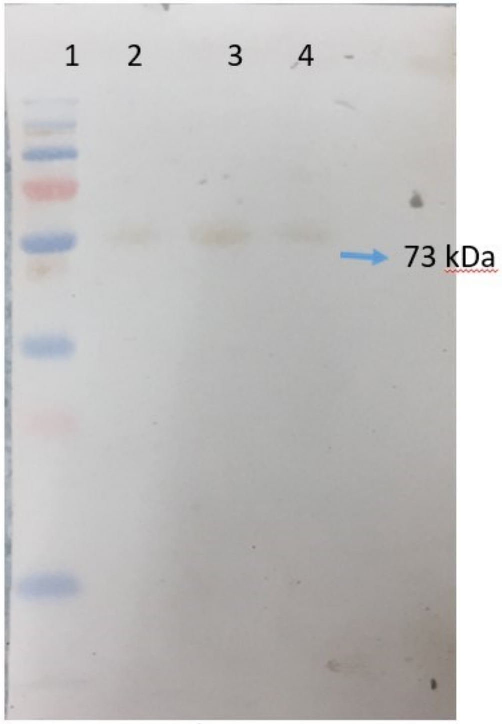

1-Marker
2-Healthy animal
3- SCM
4-CM
